# Effect of Photobiomodulation in Suppression of Oxidative Stress on Retinal Pigment Epithelium

**DOI:** 10.3390/ijms23126413

**Published:** 2022-06-08

**Authors:** Jongmin Kim, Jae Yon Won

**Affiliations:** 1 Department of Mechanical Engineering, Pohang University of Science and Technology (POSTECH), Pohang 37673, Korea; mandarinbear@postech.ac.kr; 2 Department of Ophthalmology and Visual Science, Eunpyeong St. Mary’s Hospital, The Catholic University of Korea, Seoul 03312, Korea; 3 Catholic Institute for Visual Science, College of Medicine, The Catholic University of Korea, Seoul 14662, Korea

**Keywords:** photobiomodulation, oxidative stress, antioxidant, retinal pigment epithelium degeneration

## Abstract

As the world undergoes aging, the number of age-related diseases has increased. One of them is disease related to retinal pigment epithelium (RPE) degeneration, such as age-related macular degeneration, causing vision loss without physical damage in the ocular system. It is the leading cause of blindness, with no cure. Although the exact pathogenesis is still unknown, the research shows that oxidative stress is one of the risk factors. Various molecules have been reported as anti-oxidative materials; however, the disease has not yet been conquered. Here, we would like to introduce photobiomodulation (PBM). PBM is a non-invasive treatment based on red and near-infrared light and has been used to cure various diseases by regulating cellular functions. Furthermore, recent studies showed its antioxidant effect, and due to this reason, PBM is arising as a new treatment for ocular disease. In this study, we confirm the antioxidant effect of PBM in retinal pigment epithelium via an RPE model with hypoxia. The function of RPE is protected by PBM against damage from hypoxia. Furthermore, we observed the protective mechanism of PBM by its suppression effect on reactive oxygen species generation. These results indicate that PBM shows great potential to cure RPE degeneration to help patients with blindness.

## 1. Introduction

The retinal pigment epithelium (RPE) is the polarized monolayer that lies on the Bruch’s membrane and interacts with the choroid and retina to maintain homeostasis of the ocular system. It is the base unit of the molecular transportation control system called the outer blood–retinal barrier [1,2]. In addition, it removes waste materials from the photoreceptors by phagocytosis to support the renewal of the outer photoreceptor segments [3]. Furthermore, the RPE secretes the enzyme called RPE65 to recycle retinol for phototransduction [4]. These characteristics facilitate the RPE as an essential mediator for the ocular system.

Due to this reason, the dysfunction or degeneration of the RPE cause malfunctions in the ocular system and leads to diseases related to blindness, such as age-related macular degeneration. These kinds of diseases are a global leading cause of blindness in the elderly [5,6,7]. A serious problem is that the disease occurs in the elderly, despite no physical damage to the RPE. Unfortunately, there is no cure, and the exact pathogenesis is still unknown.

Several factors, including age, race, and genetics, are reported as risk factors of RPE degeneration. Among them, the research has focused on oxidative stress [8,9]. Oxidative stress has been involved in numerous diseases, including cardiovascular and neurological disease [10,11]. Recent studies showed that this stress also affects the ocular system and might cause various ocular diseases related to retinal degeneration [12,13,14]. In particular, oxidative stress could be applied without physical damage, such as long blue light exposure, and increase the inflammatory reaction in the RPE [15]. The continuous inflammatory environment could affect the functions of the RPE and lead to damage to the other part of the retina, such as the photoreceptors [16]. Furthermore, oxidative stress could be applied through personal behavior habits such as smoking or a high-fat diet [17,18].

Various molecules, known as an antioxidant, including N acetylcysteine, ascorbic acid, and resveratrol, have been reported as treatments or prevention of RPE degeneration, and these molecules are widely consumed as health supplements. However, the effect of these molecules is still controversial. Moreover, the number of patients is expected to rise, even though various antioxidant-related health supplements are commercially available. These desperate conditions increased the demand for new treatment.

Therefore, various strategies are used to cure the disease [19,20]. Among them, a promising treatment is photobiomodulation (PBM), also known as low-level laser therapy [21]. PBM is a light-based therapy using a spectrum with a 500–1000 nm wavelength [22]. According to several studies, PBM has several effects, such as anti-inflammatory and anti-oxidative effects and cell repair [23]. With these functions, the primary advantage of PBM is that it is a non-invasive approach that facilitates the treatment of various diseases such as wound healing, stroke, peripheral nerve injury, and even heart attack [21,24]. Furthermore, the development of a light emitting diode (LED) has been applied in PBM development, which facilitated the optimization of parameters, including wavelength and irradiance time, for target diseases [25,26,27]. Given these developments, recent research has been conducted to use PBM as a new approach to cure ocular disease related to oxidative stress since it is known to prevent the production of oxidative stress molecules [28,29,30]. However, most of the research was conducted via animal models, which differ from the actual human body system and could not provide the exact curative mechanism of PBM. To evaluate the effect, safety, and mechanism of PBM, research has to be conducted with a human-based model.

Previously, we developed an RPE model using Bruch’s membrane-derived bioink [31]. The model showed the various RPE functionalities that the conventional model could not express. In this study, we developed a hypoxia model, based on our previous model, to mimic the RPE degeneration condition induced by oxidative stress. We confirmed that our model underwent hypoxia, and oxidative stress was induced. Based on this model, we evaluated the safety, effect, and curative mechanism of the PBM and confirmed that the treatment could protect RPE against damage of hypoxia.

## 2. Results

### 2.1. Development of an LED Device for Photobiomodulation

**Figure 1:**
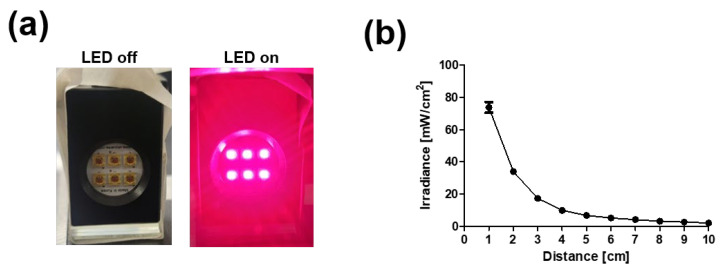
Development of light emitting diode (LED) device for PBM. (**a**) Photograph image of array of chip LED module. (**b**) Irradiance power of device vs. distance.

Six-chip LED modules with a 660 nm wavelength were combined to induce PBM, and the irradiance was inversely proportional to the distance (Figure 1a,b). Further studies were conducted at a distance of 6 cm according to previously reported research [28].

### 2.2. Hypoxia Model Development

The hypoxia condition was induced via treatment of cobalt chloride [32]. The normal RPE model was developed using a Bruch’s membrane-derived extracellular matrix-coated Transwell according to previous research [31]. Then, 200 μm of cobalt chloride was treated to induce the chemical hypoxia condition. Treatment with cobalt chloride did not affect the viability of the RPE (Figure S1a). However, the hypoxia condition was formed by the observation of hypoxia inducible factor 1a (HIF-1a, Figure S1b), which is the marker of hypoxia. Therefore, further experiments were conducted with a cobalt chloride-based hypoxia model.

### 2.3. Barrier Function

**Figure 2:**
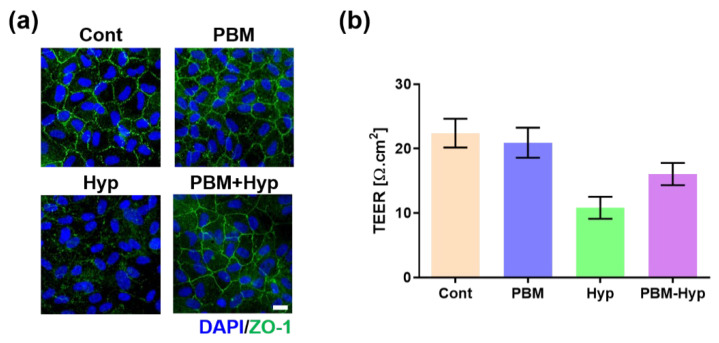
Effect of PBM on barrier function. (**a**) Immunofluorescence image of ZO-1. (**b**) TEER value. Scale: 20 μm. Cont: control model, PBM: PBM-treated model, Hyp: hypoxia model, PBM+Hyp: hypoxia model with pre-treatment with PBM. *n* = 3.

The barrier function was evaluated via ZO-1, a marker of tight junction, expression, and TEER measurement. The RPE with and without PBM treatment formed a tight junction (Figure 2a); however, its junction was disrupted in the hypoxia model. On the other hand, the hypoxia model with pre-PBM treatment showed a clear ZO-1 signal, which means there was a protection effect of PBM in the barrier function. These results were also observed in the TEER value (Figure 2b). Comparing the control and PBM-treated RPE, the TEER value was decreased in the hypoxia model, whereas pre-PBM treatment protected TEER against hypoxia damage.

### 2.4. Clearance Function

**Figure 3:**
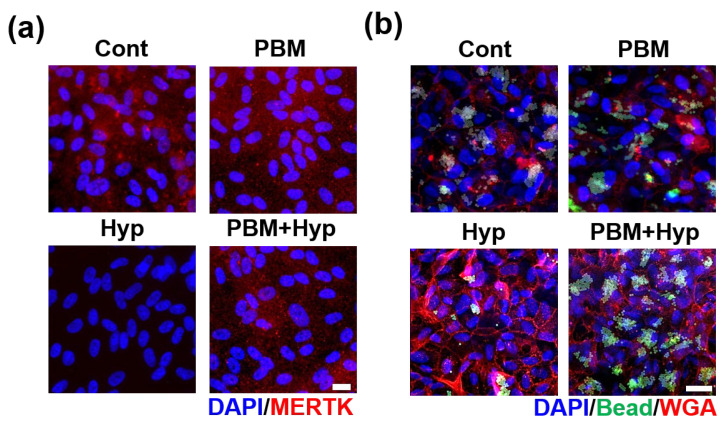
Effect of PBM in clearance function. (**a**) Immunofluorescence image of MERTK. (**b**) Bead digestion ability. Scale: 20 μm. Cont: control model, PBM: PBM-treated model, Hyp: hypoxia model, PBM+Hyp: hypoxia model with pre-treatment with PBM.

The clearance function was evaluated via MERTK expression and digestion of polystyrene beads. The expression of MERTK was quiescent in hypoxia condition, but the signal was expressed in the control, PBM, and hypoxia model with pre-treatment with PBM (PBM+Hyp) (Figure 3a and Figure S2a). Similarly, the digestion ability was decreased in the hypoxia model compared with the control, PBM-treated RPE, and pre-treatment with PBM (Figure 3b and Figure S2b).

### 2.5. Visual Function

**Figure 4:**
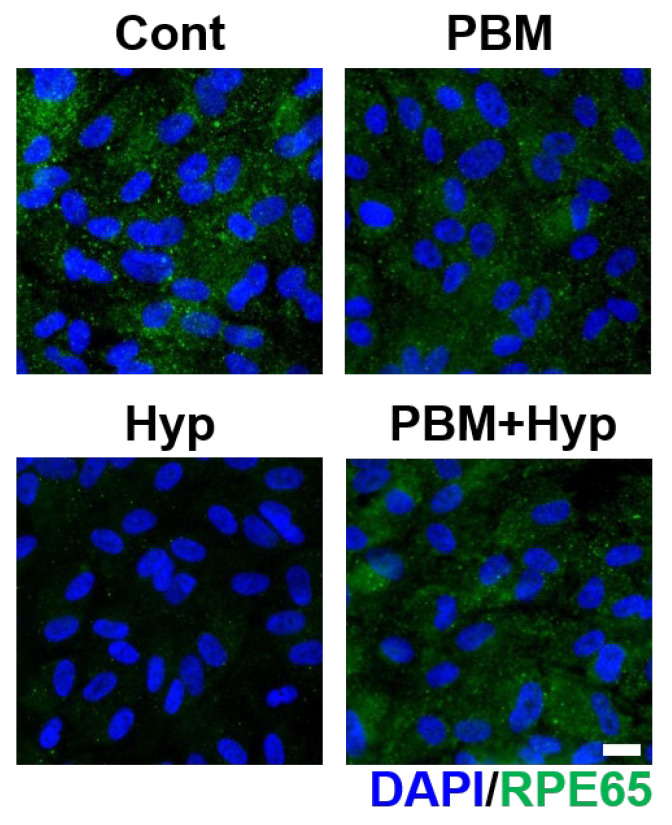
Effect of PBM in visual function. Immunofluorescence image of RPE65 enzyme. Scale: 20 μm. Cont: control model, PBM: PBM-treated model, Hyp: hypoxia model, PBM+Hyp: hypoxia model with pre-treatment with PBM.

The RPE conducted visual function by secreting an enzyme called RPE65 to support phototransduction. The visual function of RPE was examined via expression of RPE65, the enzyme for phototransduction. The control, PBM, and PBM+Hyp model exhibited expression of RPE65. However, the enzyme formation was nearly undetectable in the hypoxia model (Figure 4 and Figure S2c).

### 2.6. Oxidative Stress

**Figure 5:**
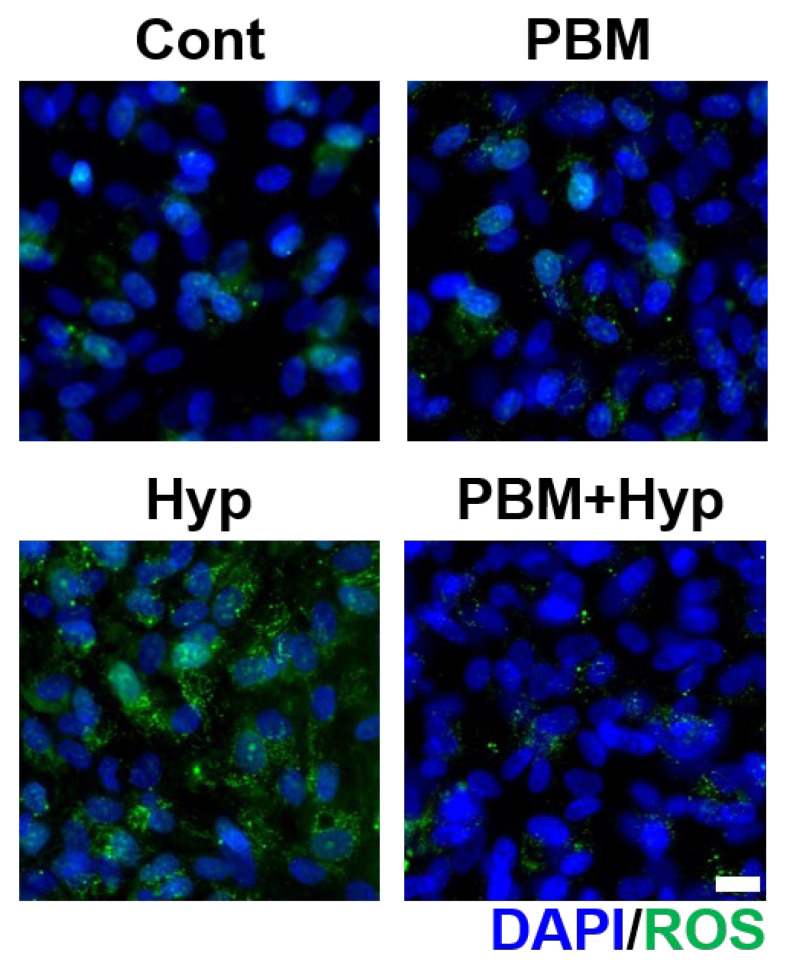
Effect of PBM on ROS generation. Immunofluorescence image of ROS. Scale: 20 μm. Cont: control model, PBM: PBM-treated model, Hyp: hypoxia model, PBM+Hyp: hypoxia model with pre-treatment with PBM.

The oxidative stress was compared via ROS generation (Figure 5 and Figure S2d). The PBM treatment did not induce oxidative stress since its ROS signal was shown to be similar to untreated RPE (control). In the case of the hypoxia model, an ROS signal was generated, which means the oxidative stress was applied in this model. The generation of ROS was prevented by pre-treatment with PBM.

## 3. Discussion

The global age of society has increased, and the number of age-related diseases has also increased accordingly. Among them, retinal degeneration is one of the most critical diseases since it affects human vision, which lowers quality of life. This disease is the leading cause of blindness, with no cure. Recent studies have focused on photobiomodulation (PBM) as a new treatment for retinal degeneration. PBM has been widely used to cure various types of tissues due to its anti-inflammation and antioxidant effect. Due to this reason, recent research has attempted to cure RPE degeneration and observed the curative effect of PBM [29,33]. However, most studies were conducted via animal models, which have different ocular characteristics than humans [34]. Even though some pilot research was performed with patients, it is hard to obtain a sample to understand the curative mechanism of PBM [35]. Due to the above limitations, a functional human-based in vitro model is required for further analysis.

Recently, various kinds of in vitro models have been widely used in the pharmaceutical industry for the drug development and research field for understanding the pathogenesis of the disease [36,37]. In particular, advanced tissue-specific ECM materials have been accelerated to develop an organ model by recapitulating the microenvironment of the target organ. Kong et al. developed a nerve-derived ECM to maturate the nerve cell with an elongated structure [38]. Lee et al. used liver-derived ECM and showed that it enhanced the functionalities of the liver cell line [39].

Previously, we developed ECM material from Bruch’s membrane (BM-ECM), the basement membrane of the RPE [31]. The BM-ECM enhances the RPE functionality by recapitulating the natural environment of the ocular system. We developed a normal RPE model via integration of BM-ECM and a commercial Transwell system and confirmed the function of RPE, including barrier, clearance, and visual functions in the developed model.

Furthermore, we developed a hypoxia model using cobalt chloride treatment. Hypoxia is one of the risk factors of RPE degeneration. We treated the cobalt chloride in our RPE model and confirmed that the model was in a hypoxia condition via expression of HIF-1a, the hypoxia marker. We further analyzed the developed hypoxia model in the aspect of RPE functionalities.

The RPE is the main unit of the outer blood–retina barrier (BRB) and interacts with the choroid and retina to maintain the homeostasis of the ocular system. The primary function of RPE is the formation of an outer BRB to control the molecular transportation from choroid to retina. The free transportation of molecules via the gap of the plasma membrane is prevented due to the tight junction. The hypoxia model showed a destructed tight junction and decreased TEER value, indicating the breakdown of the outer BRB, which is commonly presented in RPE degeneration, such as age-related macular degeneration (AMD).

The RPE supports the photoreceptors by removing molecular waste via phagocytosis of the photoreceptor outer segment (POS). This clearance function supports the function and viability of photoreceptors, and dysfunction of the clearance could damage the photoreceptors [40]. This phenomenon could be observed in the Royal College of Surgeons rat, which has a genetic disorder in the clearance function of RPE that leads to the degeneration of photoreceptors [41]. In addition, the dysfunction of phagocytosis could lead to the accumulation of the drusen, resulting in the pathogenesis of AMD [42]. The hypoxia condition affected the clearance function due to the lowered expression of MERTK, the marker of phagocytosis, as well the digestion ability of the polystyrene beads.

The secreting enzyme for the visual cycle, called RPE65, is also an essential function for phototransduction. This enzyme supports the signal process that transfers incoming light to the electric signal. Malfunction in secreting RPE65 leads to severe blindness, such as retinitis pigmentosa [43]. The secretion of RPE65 is decreased in the hypoxia model, meaning lowered visual function. The developed hypoxia model showed destructed RPE functionalities. These results showed that the hypoxia condition damaged the RPE and could increase the risk or progression to RPE degeneration.

Furthermore, we also observed that the developed hypoxia model suffered from oxidative stress. Oxidative stress has been considered an important mechanism of RPE damage in the pathogenesis of diseases related to RPE degeneration. We observed the generation of ROS, the highly reactive chemical that damages the organ, in the hypoxia model and confirmed that this phenomenon is one of the factors that damages the function of the RPE.

Based on these characteristics, we analyzed the PBM via RPE functionality changes. The pathogenesis of diseases related to RPE degeneration has been known to be associated with dysfunction of the RPE due to an increase in oxidative stress, mitochondrial dysfunction, and complement dysfunction. Several studies have presented that PBM improved disease symptoms in the disease model [34,35]. However, there is no study about the change in RPE functions, which is essential for the pathogenesis of RPE degeneration after treatment with PBM. We focused on the safety and effectiveness via treatment with PBM in normal and hypoxia models. The safety of PBM was confirmed through the expression of similar functions between the PBM-treated RPE model (PBM) and the non-treated model (control). In addition, the effect of PBM against hypoxia was confirmed since the hypoxia model with pre-treatment of PBM (PBM+Hyp) showed the protected barrier, clearance, and visual functions of RPE.

We further analyzed the protection or curative mechanism of PBM in the context of ROS generation. We observed that the hypoxia condition generated the ROS and confirmed that this condition damaged the RPE, leading to it losing its natural functionalities. PBM is known to prevent ROS generation, and we confirmed this suppression effect via our hypoxia model. The PBM pre-treated hypoxia model did not show ROS generation, whereas the hypoxia model showed elevated ROS levels. These results show that our hypoxia model reflected the physiological condition of hypoxia-induced RPE degeneration; thus, the safety and effect of PBM obtained from our model could be considered close to physiological response. The above verification supports that the PBM could be applied as a practical clinical application. Since the safety and effectiveness have been confirmed, direct irradiation into the ocular system could be allowed. Due to this reason, devices with various configurations, including auto-focusing, irradiation power, wavelength, and beam path, could facilitate the optimization of effective treatment for various kinds of ocular diseases.

The above results show the versatility of our model for developing new treatments or personalized medicine. Various cures, including small molecules, antibody, gene therapy, and physical treatment, are under development for ocular disease. The developed model shows the physiological response of the human RPE and could be used as an evaluation of the cure in terms of safety and effectiveness. In addition, using patient-derived cells, a personalized in vitro model could be developed that could be used for personalized drug screening. Based on the results of our studies and the future perspective of the developed RPE model, we expect that PBM could improve several dysfunctions of the RPE and have potential as a new therapy for RPE degeneration.

## 4. Materials and Methods

### 4.1. Light-Emitting Diode (LED) Device Development

The 6-chip LED modules were arrayed and combined with variable resistance controllers (Skycares, Gimpo, Korea). The irradiance of the device was measured with a radiation recorder (TES-1333R, TES Electronic Corp, Taipei, Taiwan) in a dark room.

### 4.2. Bruch’s Membrane-Derived ECM (BM-ECM) Preparation

The BM-ECM was prepared according to previous research [31]. Briefly, porcine eyes were obtained from a local slaughterhouse within 1 h of scarification. The surrounding tissue was detached and the eyes were hemisected. Then, the cornea, vitreous body, and retina were peeled off to obtained the RPE-BM-choroid. The collected samples were treated with 1% sodium dodecyl sulfate (SDS) solution to detach the RPE and choroid layers. The cleaned BM was washed with distilled water, lyophilized, and stored at −20 °C. For digestion of BM-ECM, 10 mg of lyophilized BM-ECM was dissolved in 3% acetic acid with 1 mg of pepsin (Sigma, St. Louis, MO, USA) for 72 h with constant stirring.

### 4.3. Cell Culture

The RPE cell line (ARPE-19, Passage 20, ATCC) was cultured in DMEM/F12 (Gibco, Waltham, MA, USA) with 10% FBS containing N1 supplement (Sigma-Aldrich， St. Louis, MO, USA), GlutaMax (Gibco), nonessential amino acids (Gibco), taurine (Sigma-Aldrich), hydrocortisone (Sigma-Aldrich), and triiodo-thyronine (Sigma-Aldrich) on a Transwell (0.4-μm pore size, Corning Coaster, Corning, NY, USA). For RPE model development, the Transwell was coated with 100 μg/mL BM-ECM for 6 h at 37 °C, followed by washing with PBS three times. Then, 1.5 × 10^5^ cells/cm^2^ of RPE cells were seeded on the coated Transwell. The medium was changed twice a week.

### 4.4. Hypoxia Induction

The hypoxia condition was induced via cobalt chloride treatment [32]. For hypoxia model development, the cultured RPE model was treated with 200 uM cobalt chloride for 2 days. To analyze the effect of PBM, a 660 nm LED with 4 mW/cm^2^, 250 s, was used twice per day for 4 days prior to cobalt chloride treatment.

### 4.5. Viability Test

The viability of the RPE cells, depending on cobalt chloride treatment, was measured via Cell Counting Kit-8 (CCK-8, Dojindo, Rockville, MD, USA). Cells were seeded on a 96-well plate coated with BM-ECM. After treatment with cobalt chloride solutions for 2 days, the cells were washed with PBS twice and incubated with CCK-8 solution for 1 h at 37 °C. The solution was collected and its absorbance at 450 nm was measured via a microplate spectrophotometer (Molecular Devices, San Jose, CA, USA).

### 4.6. Transepithelial Electrical Resistance (TEER)

The TEER values were measured to compare the barrier function of the RPE via a Volt-Ohm meter (EVOM2, World Precision Instruments, Sarasota, FL, USA) together with an STX2 chopstick electrode. The electrode was cleaned with 70% ethanol, washed with distilled water, and incubated with pre-warmed PBS. The culture medium of apical and basal chambers was replaced with pre-warmed PBS and its resistance was measured. The TEER value was calculated by multiplying the resistance and area of the Transwell.

### 4.7. Digestion Test

To evaluate the phagocytosis activity, a digestion of polystyrene beads test was conducted. The 2 μm of fluorescence-labeled polystyrene beads (Invitrogen, Waltham, MA, USA) samples at a concentration of 1 × 10^7^/mL were treated in an apical chamber of the Transwell for 16 h. After incubation, the cells were washed with PBS three times to remove undigested beads. Then, the cells were incubated with wheat germ agglutinin (WGA) for membrane staining and the samples were washed with PBS. The washed cells were fixed with 4% paraformaldehyde (PFA) for 20 min at room temperature (RT) and treated with 5 μg/mL of wheat germ agglutinin conjugated with Alexa Fluor 594 at 37 °C for 15 min. The treated cells were washed with PBS twice and permeabilized with 0.03% Triton-X (TX) for 7 min. After permeabilization, the cells were incubated with DAPI (Invitrogen) and imaging was carried out via fluorescence microscope (Zeiss, Oberkochen, Germany).

### 4.8. Immunostaining

Cultured cells were fixed with 4% PFA for 20 min, permeabilized with 0.03% TX for 7 min, and blocked with 10% normal goat serum (Vector Laboratory, San Francisco, CA, USA) for 1 h at RT. The processed samples were then incubated with a 1:100 dilution of ZO-1, MERTK, and RPE65 antibodies overnight at 4 °C. After incubation, the samples were washed with PBS with Tween-20 (PBST) three times. The washed cells were incubated with a 1:1000 dilution of secondary antibodies conjugated with Alexa Fluor 488 or 594 (Invitrogen) and washed with PBST three times. Finally, the stained cells were co-stained with DAPI, and images of them were taken via fluorescence microscope (Zeiss). The mean fluorescence intensity of the stained samples was measured via ImageJ software (*n* = 3).

### 4.9. Staining of Reactive Oxygen Species

The ROS were stained using a CellROX oxidative stress detection kit (Invitrogen) according to the manufacturer’s instructions. The imaging was carried out via fluorescence microscope (Zeiss) and the signals were measured via ImageJ software (*n* = 3).

### 4.10. Statistical Analysis

The results were analyzed with GraphPad 8 (GraphPad Software, San Diego, CA, USA). The Student’s *t*-test was applied for mean comparisons (*n* = 3).

## 5. Conclusions

In this study, we analyzed the safety and effectiveness of PBM in RPE degeneration. For this analysis, the in vitro hypoxia model was developed and confirmed the generation of oxidative stress and related the dysfunction of the RPE, including barrier, clearance, and visual functions. Through this model, we first confirmed the safety of PBM via its non-damaging effect on the RPE. Then, its effectiveness in RPE degeneration was analyzed by pre-treatment of PBM in a hypoxia model via protected RPE functionalities, whereas the functions were destructed in the hypoxia model. Furthermore, the suppression of ROS generation, the curative mechanism of PBM, was observed. To summarize, we confirmed that our model reflected the physiological condition of normal/retinal degeneration and conducted a pilot study for analyzing the effect of PBM as a new treatment for RPE degeneration.

## Data Availability

Not applicable.

## Supplementary Materials

The following supporting information can be downloaded at https://www.mdpi.com/article/10.3390/ijms23126413/s1.
