# Supplementary material for: Effect of Photobiomodulation in Suppression of Oxidative Stress on Retinal Pigment Epithelium
Source: Int J Mol Sci. 2022 Jun 8;23(12):6413. doi: 10.3390/ijms23126413 (PMC9224180; doi:10.3390/ijms23126413)
Supplement: Supplementary file 1 [file ijms-23-06413-s001.zip › ijms-1757666-supplementary.pdf]

Supporting Information

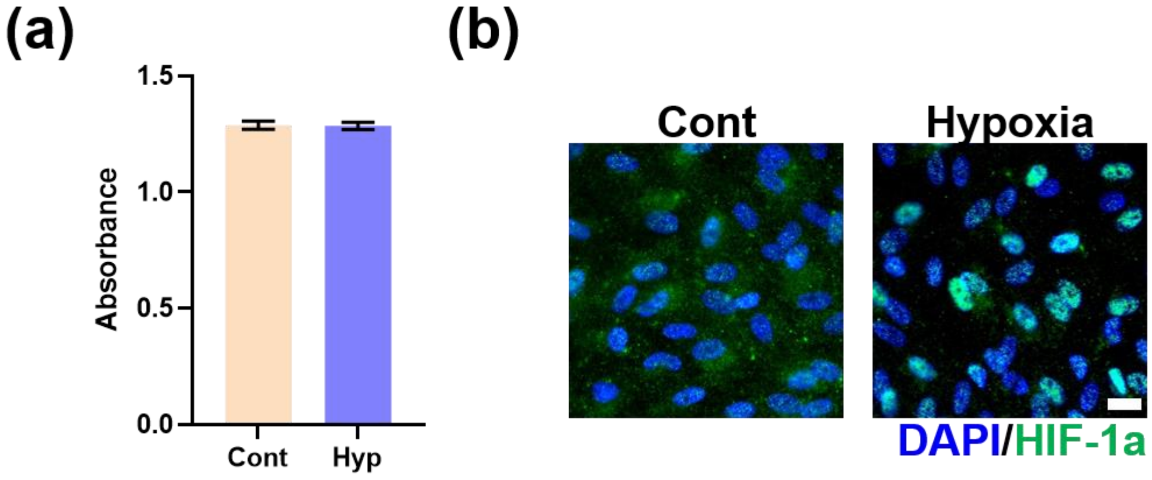

Figure S1. Development hypoxia condition via treatment of cobalt chloride. a) Viability assay. b) Expression of HIF-1a.

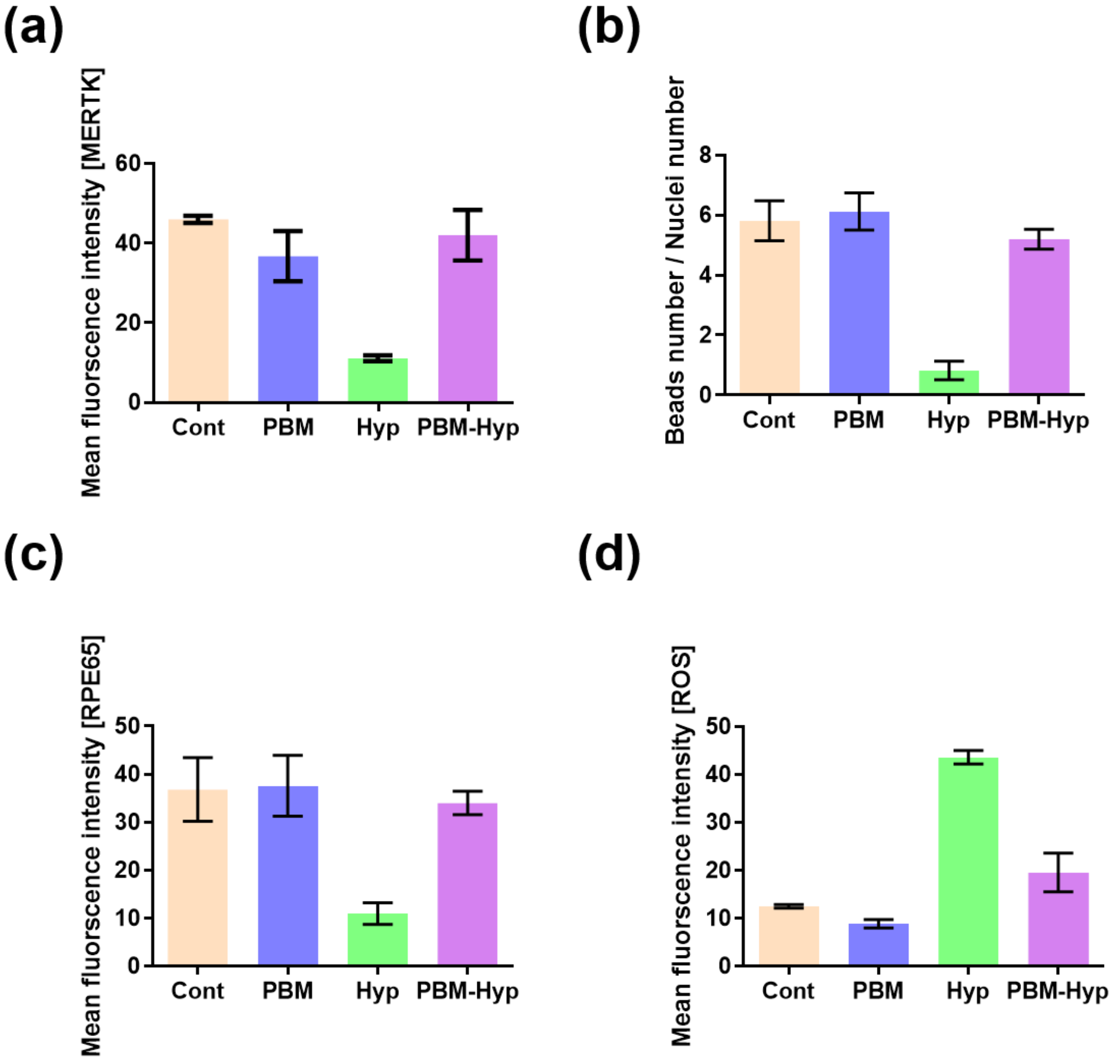

Figure S2. Comparison of RPE functionalities and ROS generation. a) MERTK. b) Digestion of polystyrene beads. c) Secretion of RPE65. d) Generation of ROS
